# Combining IR and Raman Spectroscopies for Enhanced Accuracy and Precision in the Determination of Lipid Composition in Liposomes

**DOI:** 10.3390/biom16040489

**Published:** 2026-03-25

**Authors:** Waseem Ahmed, Aneesh Vincent Veluthandath, Ganapathy Senthil Murugan

**Affiliations:** 1 Optoelectronics Research Centre, University of Southampton, Southampton SO17 1BJ, UKavv1a15@soton.ac.uk (A.V.V.); 2 Perioperative and Critical Care Theme, NIHR Southampton Biomedical Research Centre, University Hospital Southampton NHS Foundation Trust, Southampton SO16 6YD, UK

**Keywords:** machine learning, prediction intervals, data fusion

## Abstract

Reducing measurement uncertainty is crucial to enable the adoption of rapid point-of-use techniques for clinical and industrial applications. Diagnosis of neonatal respiratory distress syndrome and liposome formulation quality control are two applications for which measuring the ratio of the lecithin to sphingomyelin composition of liposomes is important, for which no rapid measurement currently exists. Raman and infrared spectroscopies are two complementary approaches to examine characteristic molecular vibrations that can spectroscopically measure liposomes and, when combined with machine learning, predict their composition. We show that employing a data-fusion approach the uncertainty in the predicted compositions compared to the individual modalities (IR *R*^2^: 0.902 and Raman *R*^2^: 0.951) can be reduced to obtain more accurate and precise measurements (low-level fused model *R*^2^: 0.973, mean squared error: 0.024, prediction interval width: 0.303, high-level weighted fusion model *R*^2^: 0.970, mean squared error: 0.027, prediction interval width: 0.268).

## 1. Introduction

Raman and infrared spectroscopy are two vibrational spectroscopic approaches to determine fundamental vibrational modes in molecules. Raman spectroscopy is based on the frequency shift of a scattered photon corresponding to the difference in the vibrational frequency of the molecular mode when compared to the input photon, and the magnitude of the interaction is measured as a change in the molecular polarisability. Infrared spectroscopy is based on different selection rules for the absorption of incident electromagnetic radiation, corresponding to the fundamental molecular vibrational modes. The spectra generated by both approaches can be used to identify and quantify chemical constituents present in a sample. The different selection rules give rise to unique spectral responses for each modality, and infrared spectroscopy is more difficult within a water matrix due to its strong absorbances in the 3200 to 3600 cm^−1^ (OH stretch vibrations), ~1645 cm^−1^ (OH bend vibrations) and 800 cm^−1^ (librational modes of water) regions [1], which can obscure analyte peaks and reduce spectral contrast. Raman spectroscopy is not so affected because water is a weak Raman scatterer. Similarly, Raman spectroscopy is impacted by fluorescence of the sample or the substrate, which adds complexity to the spectrum and hence makes it more challenging to interpret, while infrared spectroscopy typically does not suffer from these effects. Together, the two approaches are complementary [2] and so give rise to additional spectral features that can contribute to multivariate modelling of concentration-related parameters, so combining their strengths can give rise to better measurements, particularly when measuring samples in clinical contexts, which are usually obtained in a water matrix.

Lipids are amphiphilic molecules that self-assemble in aqueous environments to form supramolecular structures, such as micelles or bilayer-based vesicles. The physicochemical properties of these assemblies depend strongly on lipid composition, although determining the precise lipid composition of vesicles typically requires destructive analytical techniques, such as mass-spectrometry-based lipidomics. Dipalmitoylphosphatidylcholine (DPPC, the major lecithin, L, in pulmonary surfactant) is a glycerophospholipid that constitutes the structural backbone of many lipid bilayers. Due to its fully saturated palmitoyl chains, L promotes tight molecular packing and high membrane order, producing relatively rigid and mechanically stable membranes [3]. Sphingomyelin (S), by contrast, is a sphingolipid based on a sphingosine backbone containing a trans double bond. It forms extensive interactions with neighbouring lipids through hydrogen bonding and Van der Waals interactions, enabling the formation of highly ordered membrane domains commonly referred to as lipid rafts [4]. These domains contribute to membrane organisation and can influence interactions between vesicles and cellular membranes that facilitate uptake of vesicular payloads [5]. Together, L and S can form highly stable vesicles whose physicochemical properties can be tuned by altering their relative proportions, and increasing S generally increases membrane order and stability, whereas increasing L increases bilayer fluidity and vesicle–membrane fusion dynamics [6].

Measurement of lipid concentration and composition has been proposed as a useful approach for diagnosing a range of clinical conditions, including neonatal respiratory distress syndrome (nRDS) [7] and certain cancers [8]. In pulmonary surfactant biology, lipids are packaged within lamellar bodies-specialised vesicles produced by alveolar type II cells, which release large quantities of L along with other surfactant components at the alveolar air–liquid interface of the lungs [9,10]. This surfactant layer reduces surface tension to very low levels, preventing alveolar collapse and enabling efficient respiration. In premature neonates, however, the metabolic pathways responsible for producing L are not fully developed, resulting in lamellar bodies with a reduced lecithin-to-sphingomyelin (L/S) ratio. This imbalance is a primary cause of neonatal respiratory distress syndrome, a leading cause of mortality during the first month of life [11]. Clinically, an L/S ratio below approximately 2.2 is considered diagnostic of surfactant deficiency associated with nRDS. Rapid identification of this condition is important because early intervention is associated with improved clinical outcomes, yet no point-of-care diagnostic test currently exists to measure the L/S ratio directly [12].

Diagnosis of nRDS, therefore, remains challenging [13], as presenting symptoms often overlap with other neonatal pulmonary and systemic conditions and management is typically initiated on a symptomatic basis. This can delay identification of the underlying pathology and compromise the effectiveness of targeted treatment [12]. A point-of-care method capable of directly determining the L/S ratio of lamellar bodies in bronchoalveolar fluid taken from a patient would provide clinicians with rapid biochemical information to guide diagnosis and treatment decisions, potentially reducing disease severity and shortening neonatal intensive care stays. Vibrational spectroscopy represents one potential strategy for achieving rapid, minimally destructive measurement of the L/S ratio in a clinical setting.

Beyond pulmonary diagnostics, lipid vesicles, such as liposomes, are of growing biomedical importance because of their widespread use in drug delivery [5,14,15,16] and pharmaceutical formulations. Liposomes have been investigated for applications ranging from conventional drug delivery to nutrients and nucleic-acid-based therapeutics, such as mRNA and DNA vaccines [2]. Their amphiphilic bilayer structure allows them to encapsulate hydrophilic drugs within the aqueous core while incorporating hydrophobic compounds within the lipid membrane, protecting therapeutic agents from degradation and enhancing delivery to target tissues. Furthermore, the size, composition, and surface properties of liposomes can be engineered to modify circulation time, target specific tissues, and reduce toxicity. Liposomes can also fuse with cellular membranes to release their payload directly into cells, making them effective platforms for gene therapy and vaccine delivery.

Data fusion is the combining of information from discrete sources in a specified manner. The data sources may be different methods of measurement, which together provide additional features and information (such as “patient age”, “weight” or “current medications”) for modelling. Combining datasets from multiple sources has been shown to improve the reliability and accuracy of a chemometric model [17]. In the context of the methods used for establishing the L/S ratio of lipids in this study, this culminates in reduced uncertainty of machine learning models. Data fusion [18] can be performed at three distinct levels: full spectrum (low-level data fusion), feature level (mid-level data fusion), and at the decision level (high-level data fusion). For full-spectrum data fusion, once spectra have been appropriately pre-processed, the data for each sample, measured using the different modalities, are concatenated and used to train a prediction model for some response variable. Feature-level data fusion considers each dataset and applies algorithms to identify the best features from each dataset to combine and generate the prediction model, while the decision-level data fusion takes the outputs of models generated on each modality and combines them in a specified manner to give a final prediction. One of the goals of a fusion approach is to provide more accurate and precise predictions of the target analyte. Reducing variance, which represents instability and uncertainty in predictions, has been employed to improve the precision of measurements. One approach to reducing the variance [19,20], particularly in the high-level fusion scenario, is to use the variance in each sample to weigh the combination of model outputs from each modality. In doing so, this specifically emphasises the modality, which had the least uncertainty for that sample point—resulting in a reduction of the final output uncertainty [21].

Data fusion of FTIR and Raman spectra has been reported for monitoring the spoilage of beef [22] and for profiling impurities in fentanyl precursors [23], but these studies did not find significant improvement in prediction accuracy when employing the data fusion technique, likely due to a lack of optimisation. A study, which used Raman and FTIR to measure peroxide and acid values of edible oils [24], reported improvement from the individual modalities by employing a mid-level fusion approach and using a successive projection algorithm to select strong features to model. Another study [25] reported that using low-level fusion and standard normal variate (SNV) transformation of baseline corrected data, to develop a global model to detect adulterants in marine oils, performed better than other global models based on individual spectroscopic modalities. However, they also found that the models based on the infrared modality alone performed the best for detecting generic fish oil adulteration in cod liver and salmon oils, indicating that there are other strategies available to achieve better precision and accuracy with machine learning models.

In this study, vesicles were formulated as a simplified model system representing both pulmonary lamellar bodies and liposomes used in pharmaceutical formulations. The work was motivated by two complementary objectives: first, to develop an approach to rapidly diagnose nRDS through measuring the L/S ratio of lamellar bodies, and second, to investigate the capability of vibrational spectroscopy to characterise lipid compositions in liposomes used in pharmaceutical and cosmetic products.

A major barrier to clinical adoption of liposome-based nanomedicine [26] is the absence of methods to perform rapid quality and composition checks without extensive sample preparation. Vibrational spectroscopy-based methods offer a label-free, sensitive, and rapid approach to quantify lipid compositions of aqueous vesicle systems, analogous to both aqueous liposomes and lamellar bodies, without extensive sample preparation. Such methods have potential for both monitoring lipid formulations during manufacturing processes and rapid point-of-care measurement of lamellar bodies.

In this work, Raman and Fourier transform infrared (FTIR) spectroscopy were used to quantify lipid concentrations in vesicles composed of L and S, which serve as controlled physicochemical analogues of liposomes and lamellar bodies found in bronchoalveolar lavage fluid. To evaluate predictive performance, we compared independent modelling strategies using partial least squares regression, including low-level and high-level data fusion approaches, and assessed their relative accuracy and precision for determining lipid composition.

## 2. Materials and Methods

### 2.1. Sample Preparation and Measurements Methodology

DPPC (L) and S were purchased from Avanti Polar Lipids (Avanti Research, Alabaster, AL, USA). HPLC-grade dichloromethane (DCM) was purchased from Sigma Aldrich (Merck KGaA, Darmstadt, Germany). Aqueous vesicles with various L/S ratios (L/S 0.25, 0.5, 0.75, 1.00, 1.25, 1.50, 1.75, 2.00, 2.25, 2.50, 2.75, 3.00, 3.25, and pure L and S) were prepared as described by Veluthandath et al. [27]. The vesicles are stored in a refrigerator for measurement.

The samples underwent Raman spectroscopy analysis utilizing a Renishaw inVia micro-Raman spectrometer, employing a 532 nm laser for excitation. The microscope was configured in epi-fluorescence mode, with excitation through a 50× objective lens, and the resulting Raman scattered light collected through the same lens. A notch filter was incorporated to eliminate the excitation laser light from the Raman spectrum. A silicon wafer was used as a substrate on which 25 μL of the sample was deposited for recording the Raman spectrum. Raman scattering from the Si wafer was used for spectrometer calibration. Spectra were captured with a 10-s exposure time, employing five spectra averaging, and maintaining a laser power of approximately 5 mW. All measurements were conducted at room temperature. The spectra were cosmic spike corrected and normalised. The spectra were processed by normalisation using the standard normal variate (SNV).

FTIR measurements were performed on an Agilent^®^ Cary 670 FTIR instrument (Agilent Technologies, Inc., Santa Clara, CA, USA), with a potassium bromide (KBr) beam splitter, and a deuterated triglycine sulphate (DTGS) detector. This was controlled by Resolutions Pro^®^ software (v4). A 10-bounce Pike^®^ (PIKE Technologies, Inc., Madison, WI, USA) zinc selenide (ZnSe) horizontal ATR accessory with a solvent lid was used as the sample platform. Nitrogen purging was set to 9 L/min. Scan settings were set to 32 readings at a $4\;cm^{-1}$ resolution and backgrounded against pure water. Post collection, the spectra were baseline offset corrected in Peak^®^ Spectroscopy (v4.00 build 484, Peak Scientific, Glasgow, Scotland, UK) at $2500\;cm^{-1}$ and converted into CSV files for further analysis using the Python library Scikit-Learn (v1.1.3) for modelling and Pandas to perform the data fusion.

### 2.2. Prediction Interval and Figures of Merit

Prediction intervals give a range for which a future observation is likely to fall within, and is specified with respect to a given probability, α, usually taken to be 0.05, from which the prediction interval nominal coverage percentage (PINC) is defined [28]:

(1) $$PINC=100\times \left(1-\alpha \right)\%.$$

When α is taken as 0.05, this translates to intervals within which, in 95% of the predictions made by a model, the actual value will fall within and encompasses the errors present within the spectral data as well as those in the determination of the model parameters. The figure of merit was used to describe the performance of the prediction intervals, which capture the actual value of an applied analyte and begin with the prediction interval coverage probability (PICP) [29], which can be applied identically in cases where the prediction intervals are generated from parametric and non-parametric data:

(2) $$PICP=\frac{1}{N_{test}}\sum_{i=1}^{N_{test}}c_{i}$$

where

$$N_{test}=number\;of\;samples\;in\;test\;set$$

$$c_{i}=\left\{\begin{array}{c} 1,\;t_{i}\in \left[Lower\;PI\;bound,\;Upper\;PI\;bound\right]\\ 0,\;t_{i}\notin \left[Lower\;PI\;bound,\;Upper\;PI\;bound\right] \end{array}\right.$$

$$t_{i}=model\;output.$$

When the prediction intervals are specified such that the actual value of the analytes measured fall within the prediction interval range, a PICP value of one is obtained. The PICP and PINC can be combined to give a further figure of merit as the average coverage error, ACE, defined as follows:

(3) ACE=PICP−PINC

where ACE values close to zero are considered better calibrated prediction intervals [30] because they indicate that the prediction interval coverage matches the nominal coverage specified during the model generation. In contrast, positive ACE values indicate that the prediction interval is overly conservative, while negative values indicate that the intervals are too narrow and fail to properly account for the uncertainty in the data. The ACE value can therefore be used as a measure of how well the uncertainty estimated by the model reflects the true variability of the predictions. Mis-specifying prediction intervals by widening them can guarantee a full coverage of model outputs, but doing so can detract from their utility to provide useful model outputs, which can, in this context, confound their use as a guide to diagnosis or lipid composition monitoring. Narrower prediction intervals reduce the uncertainty in the precise model output, but, if incorrectly specified, have a greater chance of not covering the true analyte concentration or ratio. This effect can be quantified using the “sharpness” of the prediction interval width as the prediction interval normalised average width (PINAW) [10]:

(4) $$PINAW=\frac{1}{NR}\sum_{t=1}^{N}\left(Upper\;PI\;bound-Lower\;PI\;bound\right)$$

where

R=Range of actual values used to normalise the prediction intervals

N=Number of samples used.

Smaller PINAW values are obtained when the prediction intervals are smaller, indicating less uncertainty in the measurement, which is generally more desirable if they remain well calibrated.

### 2.3. Machine Learning Methodology

Spectra from the FTIR and Raman datasets were matched so that there were an equal number of L/S ratio datapoints from each modality. This pairing was maintained for each time the data were split into either training or test datasets, and further for the cross-validation datasets. For the low-level fusion models, both datasets were normalised, prior to row-wise concatenation, using SNV and selecting a spectral region free of any substrate or environmental character to establish the correct normalisation parameters, which are then applied across the spectrum. For the FTIR spectra, this was defined as $850–2000\;{\mathrm{cm}}^{-1}$, missing out the bands due to carbon dioxide and for the whole Raman spectra, which would have added noise to the data unconnected to the concentrations of the analytes. SNV was chosen because of its application on a single-spectrum basis, ensuring that preprocessing performed on one spectrum did not affect any other. This was used to rescale the spectra so that the peaks from each modality were approximately similar in intensity. Spectra from identical concentrations were matched for each modality and assigned an index number.

The train/test split allocated 80% of the data to the training set and 20% to the test set. Further steps were identical for all models tested. The machine learning data pathway is shown in Supplementary Materials Figure S1. This approach was utilised because it provided sufficient data for the model training and evaluating performance. The absence of the L/S=1.5  samples from the test set arising from the random train/test split in combination with the limited dataset size. Because the same test set was used as the basis for comparison across all individual and fusion models, this did not affect the fairness of the comparative assessment of modelling strategy.

The training-set data were further partitioned 80%/20% into 50 different, randomly assigned, cross-validation training and test sets, respectively. These were used to train and assess incrementally more complex partial least squared regression (PLSR) models from 1 to 30 latent variables (LVs), which were evaluated using the average mean squared error (MSE) and $R^{2}$ from the cross-validation training and test sets. The optimal model was selected based on whether the addition of a subsequent LV contributed significantly to the model performance, based on the cross-validation performed, by comparing the F-statistic for a less complex model to the model that provided the lowest MSE [14] in the cross-validation test set. This usually results in models with fewer LVs than models selected based on a minimum MSE, which, due to their more parsimonious nature, tend to perform better on new data. Once the appropriate model complexity was determined, the model was retrained using the full training set and used to predict the L/S ratio of independent test set spectra.

The high-level weighted fusion model weighted the contribution from each modality by considering the prediction interval range as a proxy for the prediction uncertainty (z=1.96 for 95% prediction intervals (PI)):

(5) $$\sigma \approx \frac{Upper\;PI-lower\;PI}{2z}.$$

The weighting applied to each modality was then calculated using inverse variance weighting:

(6) $$weight\;(w)=\frac{1}{\sigma^{2}}.$$

To fuse the outputs of each model, the weighted prediction was calculated as follows:

(7) $$Prediction=\frac{\left(w_{FTIR}\times Prediction_{FTIR})+{(w}_{Raman}\times Prediction_{Raman}\right)}{w_{FTIR}+w_{Raman}}.$$

The prediction interval width was used as a proxy for prediction uncertainty because the prediction intervals were generated using a conformal prediction approach. Conformal prediction provides distribution-free uncertainty estimates with guaranteed coverage but does not directly provide a parametric estimate of prediction variance.

The performance of each of the models was evaluated using the $R^{2}$ of test set predictions and with reference to the uncertainty intervals provided by the model as measures of accuracy and precision. The purpose of this study was to understand how two different fusion strategies affect the uncertainty in the regressed prediction from spectra in a test set. The spectra comprising the training, cross-validation and test sets used for each model had identical index numbers, and the models were trained on the full spectral range collected.

## 3. Results and Discussion

### 3.1. Impact of Water on Raman and FTIR Spectra

**Figure 1:**
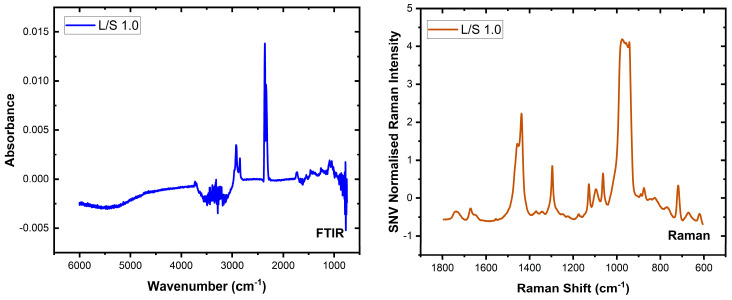
Representative FTIR (**left**, blue) and Raman (**right**, orange) spectra showing the spectra recorded from vesicle samples, in a water matrix, with an L/S ratio of 1.0.

Water is a strong absorber of infrared light and has features that can be readily observed in the mid-infrared spectrum due to the strong absorption by the OH stretch (near 3200 to 3600 cm^−1^) and bend vibrations (near 1645 cm^−1^). Figure 1 (left) shows one infrared spectrum recorded in this study for an L/S ratio of 1.0 and demonstrates the impact of liquid water on the spectrum of lecithin and sphingomyelin, which have features in both regions. This manifests as a negative peak in the 3200 to 3600 cm^−1^ region after background correction, and is attributable to a mismatch between the background and sample absorptions. Similarly, around 1645 cm^−1^ and 800 cm^−1^ negative peaks attributable to regions of strong water absorption are co-located with regions in which lipid molecules exhibit peaks. Figure 1 (right) shows the Raman spectrum of the same sample, free of any negative peaks. In contrast to FTIR spectroscopy, Raman spectra are less affected by water. However, Raman measurements often contain contributions from the substrate and can be obscured by fluorescence from the sample, solvent, or substrate. Strong Raman bands from the substrate or solvent can also be problematic. For example, in Figure 1 (right), which shows the Raman spectrum of the sample with an L/S ratio of 1.0, the strongest feature in the 920–1000 cm^−1^ range arises from the 2TO vibration of the silicon substrate. In addition, fluorescence from the sample or substrate can be more intense than Raman scattering, thus masking the Raman peaks.

Despite these limitations, the two spectroscopic techniques provide complementary information. In this study, bands associated with the phosphate group are clearly visible in the FTIR spectra but are not observed in the Raman spectra (see Figure S2 in the Supplementary Materials). Conversely, Raman spectroscopy provides spectra largely free from the strong water absorption artefacts observed in FTIR measurements. Combining the two modalities, therefore, allows spectral information that is obscured or weak in one technique to be recovered from the other, providing a more complete representation of the lipid composition of the vesicle system.

### 3.2. Analysis of Raman and FTIR Spectra

**Figure 2:**
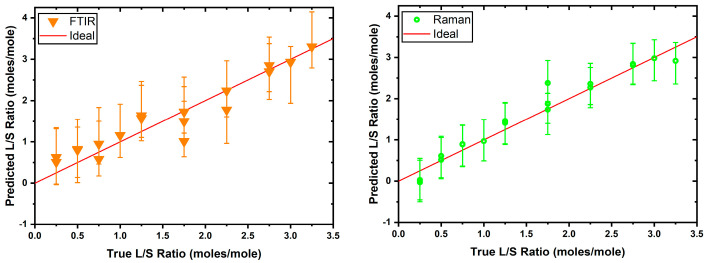
Test set performance for the FTIR (**left**) and Raman (**right**) PLSR models. The red line indicates the ideal prediction for each L/S ratio across the tested range. The absence of points around the L/S 1.5 is due to the absence of this data point in the test set, due to the random nature of the train/test split.

The first set of models based on the FTIR and Raman datasets (see Figure 1) was independently used to establish a baseline performance for comparison against the fused models. To this end, the FTIR and Raman PLSR models were trained as detailed above, and it was established that the FTIR model was optimally described by a five LV model ($R_{train}^{2}:0.927,\;R_{test}^{2}:0.902,\;mean\;squared\;error\left(MSE\right):0.0847$), while the Raman model by eight LVs ($R_{train}^{2}:0.991,\;R_{test}^{2}:0.951,\;MSE:0.0622$). The 95% prediction intervals were generated using the jackknife+-after-bootstrap method [31], which is a model-agnostic method that can be applied to data that do not necessarily follow a parametric distribution, and the test set performance of these models was evaluated as shown in Figure 2.

The high-test set $R^{2}$ values (FTIR $R^{2}$: 0.902, Raman $R^{2}$: 0.951) for each model indicate a well-specified model for each modality, although between the two, the Raman model was notably better. This was likely due to many features in the mid-IR spectrum being obscured by strong water absorption, which is dominant in this spectral region.

### 3.3. Fusion of Data

**Figure 3:**
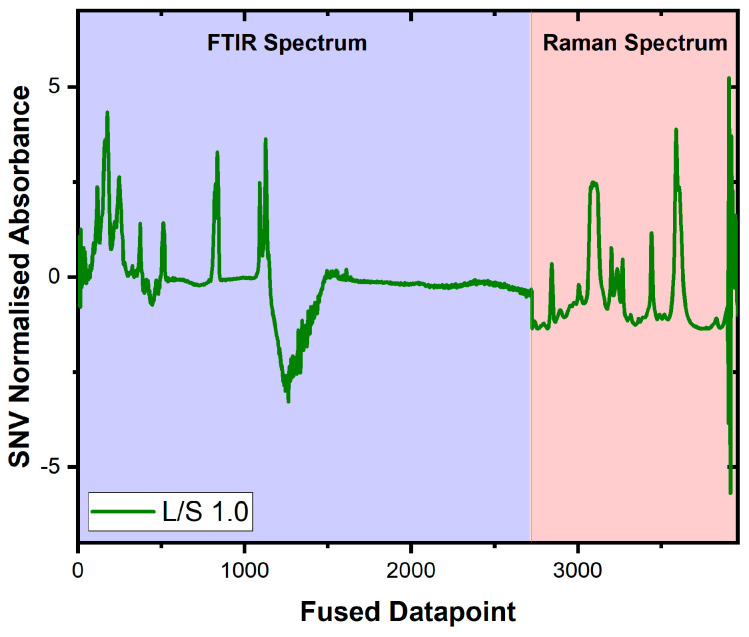
Sample spectrum of low-level fused FTIR and Raman data showing the results of SNV normalisation of both spectra and then concatenating the data matrices.

**Figure 4:**
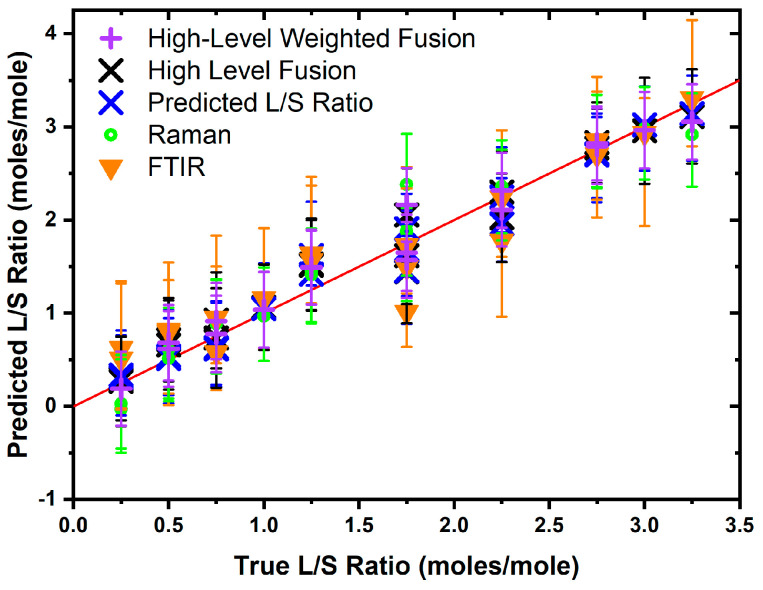
Test set performance of different models. The red line indicates the ideal prediction for each L/S ratio across the tested range. The absence of points around L/S of 1.5 is due to the absence of this data point in the test set, due to the random nature of the train/test split.

The high-level fusion model (see Figure 3) was specified as the mean output from both the Raman and FTIR models, and the consequent test set $R^{2}$ performance (${\;R}_{test}^{2}:\;0.967,\;MSE:0.0622)$ was better than both. The low-level fusion concatenates the dataset for the FTIR and Raman spectra (see Figure 4) prior to training a separate PLSR model, and this was achieved by applying the SNV to both the FTIR and Raman spectra prior to concatenation. The row-wise concatenation results in the generation of a common fused datapoint axis, formed of sequential data points in each set of spectra, as opposed to the wavenumber or Raman shift scale, which could directly be interpreted as molecular vibrational modes. The low-level fusion model was trained on the same training data used in training the individual models, which determined that ten LVs ($R_{train}^{2}:0.990,{\;R}_{test}^{2}:0.973,\;MSE:0.0343$) were adequate to model the data. It is important to note that low-level fusion performed in this manner does not guarantee that both modalities contribute equally to the predicted output, but by adopting a PLSR approach, the latent variables constructed maximise the covariance between the spectra and the L/S ratio. This means that the spectral regions most associated with variation in the L/S ratio contribute more strongly to the model. The $R_{test}^{2}$ values of the low-level fusion model were larger (the MSE values were lower) than those for the individual and high-level data fusion models, indicating that this model more accurately predicted the L/S ratio for the test set data (see Figure 4). A prediction interval-aware method of combining the two modalities was employed to generate a high-level weighted fusion model. This approach estimates the variance associated with each modality and applies weights proportional to the inverse of this variance, giving greater influence on the modality with lower prediction uncertainty. The high-level weighted fusion model was slightly less accurate than the low-level fusion model (${\;R}_{test}^{2}:\;0.970,\;MSE:0.0270)$.

The better performance of the low-level model than either of the two individual modality models supports the proposition that fusion models can be more accurate than individual modalities. The low- and high-level fusion models’ performance was more accurate than the Raman model when assessed using the test set MSE, where the smaller values obtained from the fusion models are evidence of an increase in accuracy. The prediction interval performance (see Table 1) for each of the models shows that the high-level weighted fusion model had the lowest PINAW value (0.268), followed by the low-level fusion model (0.303), which outperformed the high-level fusion model (0.324), the Raman model (0.333) and the FTIR model (0.450). This shows that the inverse variance weighting approach can effectively combine individual model outputs and provide a more precise and less uncertain final output prediction. The high-level weighted fusion, low-level and Raman models have an ACE value closer to zero, which would suggest that the prediction intervals are well calibrated for the nominal uncertainty specified (PINC=95%). The FTIR and high-level fusion models both have ACE values of 0.050, which indicates a possibility that the prediction intervals for these models may be overly conservative, but the limited size of the dataset precludes the possibility of making firm conclusions in this regard. With consideration to the $R^{2}$, PINAW, and ACE values, the low-level fusion model provides the highest predictive accuracy (lowest MSE) while the high-level weighted fusion model provides the greatest precision through narrower prediction intervals (smaller PINAW).

It is likely that the models featured within this study could be further improved by performing feature selection within each spectral dataset to establish those wavenumbers which best model the L/S ratio changes in the data, and then to build a model with these selected datapoints. This would reduce the complexity in the dataset whilst retaining the critical information required for calibrating the machine learning models, in line with an ideal of a parsimonious model, which is where a simpler model is deemed better for modelling a scenario when a simple and complex model both exist, as it has inherently better generalisability. While in general model parsimony is useful for generating calibration curves for analyte concentration based on absorbance data, there are cases when a more complex model is better suited to a task because of the greater resilience against interferents and noise.

## 4. Conclusions

Vibrational spectroscopy is a fast, label-free method to analyse aqueous vesicles. We have explored the feasibility of using both FTIR and Raman spectroscopy along with machine learning to determine the ratio of lipids in aqueous vesicles. The test sets of both FTIR ($R^{2}$: 0.902, MSE: 0.089) and Raman ($R^{2}$: 0.951, MSE: 0.0445) show high $R^{2}$ and low MSE, indicating well-specified models with good accuracy. The high-level fusion model showed an improved test set $R^{2}$ and MSE performance ($R^{2}:\;0.967,\;MSE:0.030)$ compared to the individual FTIR and Raman models. The low-level data fusion model ($R^{2}:\;0.973,\;MSE:0.024)$ provides better performance than the individual models as well as the high-level data fusion model. The high-level weighted fusion model ($R^{2}:\;0.970,\;MSE:0.027)$ was slightly less accurate than the low-level fusion model.

Comparison of the PINAW, ACE, and PICP values showed that the prediction intervals of the Raman model performed better than the FTIR model and that the fusion models performed altogether better than the individual models. The low-level fusion model performed better than the high-level fusion model, indicating that there was much smaller uncertainty in the low-level fusion model as compared to the high-level fusion model and both the individual models. The best precision was obtained from the high-level weighted fusion model.

The figures of merit employed for this comparison were the width of the uncertainty bands, which is a measure of precision, and the predicted output mean-squared error, which is a measure of accuracy. The results show that the low-level fusion model provided the highest predictive accuracy (MSE = 0.0242) compared to FTIR, Raman and high-level fusion models of the L/S ratio. However, the smallest prediction intervals were obtained from a high-level weighted fusion model (PINAW=0.268), demonstrating that this approach produced the most precise predictions of the models tested. This work supports the notion of using data fusion approaches for developing a rapid analytical method for analyzing vesicle lipid compositions in aqueous systems, particularly for establishing the L/S ratio. Further work will be required to validate these findings with larger datasets and complex biological samples.

## Figures and Tables

**Table 1:** Prediction interval performance summary detailing the test set MSE, PICP, ACE, and PINAW metrics for each model, as well as showing the minimum, mean and maximum prediction interval size for the test set lower and upper prediction intervals. The bold values indicate the best performance between the models.

		FTIR Model	Raman Model	High-Level Fusion Model	Low-Level Fusion Model	High-Level Weighted Fusion
Lower prediction interval	Min	0.373	0.449	0.421	0.318	0.393
	Mean	0.602	0.508	0.486	0.445	0.403
	Max	0.997	0.608	0.569	0.564	0.411
Upper prediction interval	Min	0.374	0.393	0.421	0.373	0.393
	Mean	0.748	0.490	0.486	0.465	0.403
	Max	0.974	0.543	0.569	0.595	0.411
Mean Squared Error		0.0890	0.0445	0.0300	0.0242	0.0270
PICP		1.000	0.944	1.000	0.944	0.944
ACE		0.050	−0.006	0.050	−0.006	−0.006
PINAW		0.450	0.333	0.324	0.303	0.268

## Data Availability

All data supporting this study are available from the University of Southampton repository at https://doi.org/10.5258/SOTON/D3528.

## Supplementary Materials

The following supporting information can be downloaded at https://www.mdpi.com/article/10.3390/biom16040489/s1. Figure S1: Machine learning dataflow, Figure S2: Comparison of FTIR and Raman complementary spectra, Figure S3: Single line of Figure 4.

## Abbreviations

The following abbreviations are used in this manuscript:

ATR-FTIR	Attenuated total reflectance—Fourier transform infrared (spectroscopy)
PLSR	Partial least squared regression
LV	Latent variable
PINAW	Prediction interval normalised average width
ACE	Average coverage error
PICP	Prediction interval coverage probability
DPPC	Dipalmitoylphosphatidylcholine
S	Sphingomyelin
L	Lecithin
