# Supplementary material for: Combining IR and Raman Spectroscopies for Enhanced Accuracy and Precision in the Determination of Lipid Composition in Liposomes
Source: Biomolecules. 2026 Mar 25;16(4):489. doi: 10.3390/biom16040489 (PMC13113388; doi:10.3390/biom16040489)
Supplement: Supplementary file 1 [file biomolecules-16-00489-s001.zip › biomolecules-4185906-supplementary.pdf]

biomolecules

MDPI

Article

Combining IR and Raman Spectroscopies for Enhanced Accuracy and Precision in the Determination of Lipid Composition in Liposomes

Waseem Ahmed [1,2], Aneesh Vincent Veluthandath [1] and Ganapathy Senthil Murugan [1,*]

[1] Optoelectronics Research Centre, University of Southampton, Southampton SO17 1BJ, UK; avv1a15@soton.ac.uk (A.V.V.)
[2] Perioperative and Critical Care Theme, NIHR Southampton Biomedical Research Centre, University Hospital Southampton NHS Foundation Trust, Southampton SO16 6YD, UK
* Correspondence: smg@orc.soton.ac.uk

Supplementary Information

Machine learning Dataflow

 https://doi.org/10.3390/biom16040489

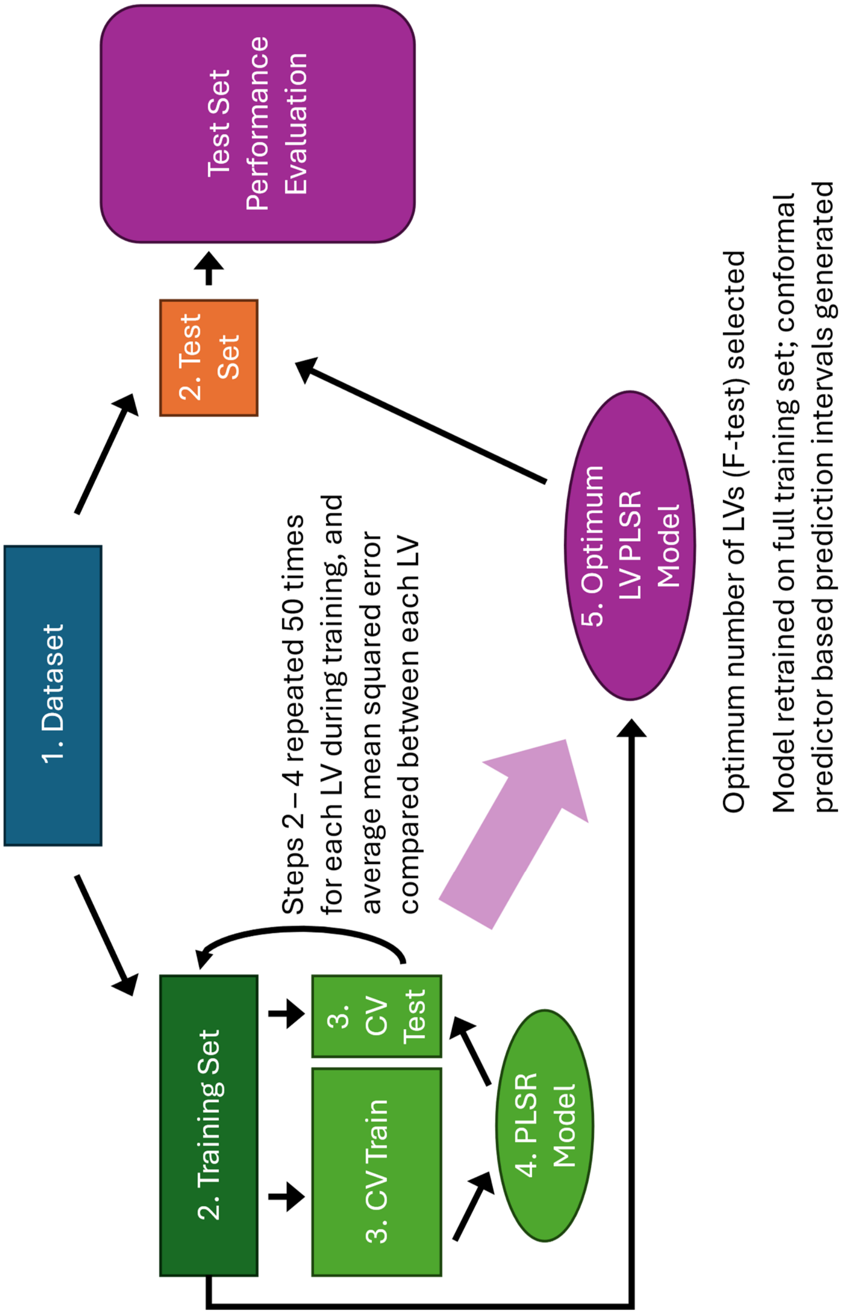

Figure S1: Machine learning dataflow showing how the data was apportioned in this study. The cross-validation approach and the method by which the prediction intervals were generated is also shown.

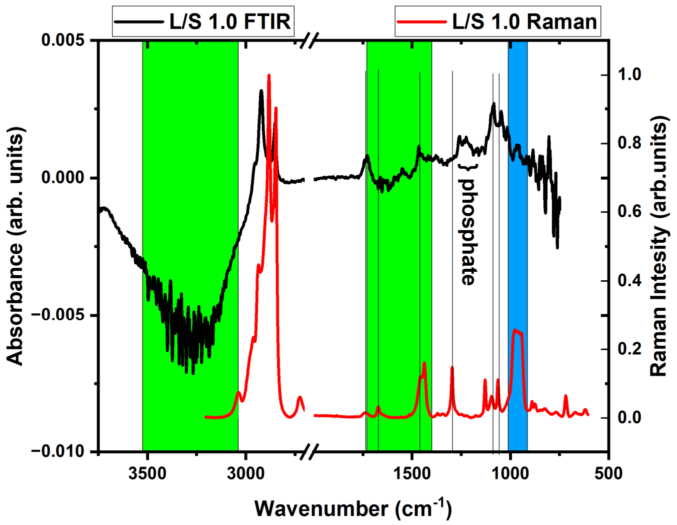

Figure S2: Graph showing complementary nature of FTIR and Raman spectra of L/S ratio 1.0 aqueous lipid vesicles. The phosphate group is indicated on the FTIR spectrum, but this feature is absent in the Raman spectrum. At the same time, the FTIR spectrum has been impacted by the aqueous solvent to the extent that negative peaks are visible in the spectrum, as indicated by the green shaded areas. In one of the areas, the Raman peaks due to DPPC C=O stretching (1737 cm^{-1}) and SM amide I (~1650 cm^{-1}) is very clear. The blue shaded area shows a peak which is related to the silicon substrate used in the Raman measurements and not a feature in the lipid Raman spectrum.

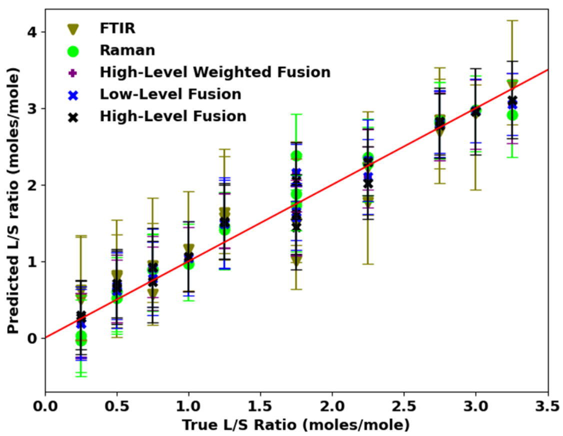
FTIR
Raman
High-Level Weighted Fusion
Low-Level Fusion
High-Level Fusion
Predicted L/S ratio (moles/mole)
True L/S Ratio (moles/mole)

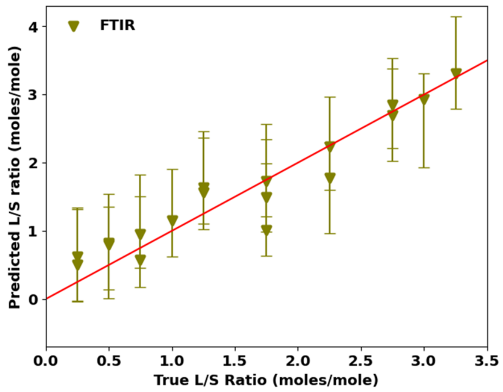
FTIR
Predicted L/S ratio (moles/mole)
True L/S Ratio (moles/mole)

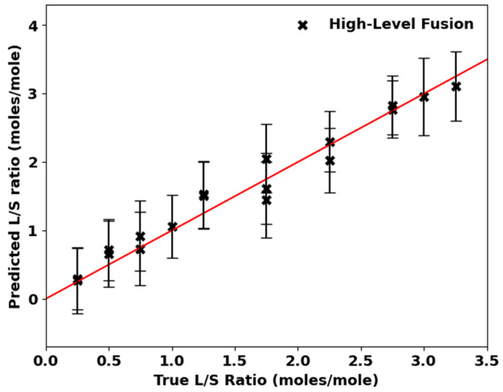
High-Level Fusion
Predicted L/S ratio (moles/mole)
True L/S Ratio (moles/mole)

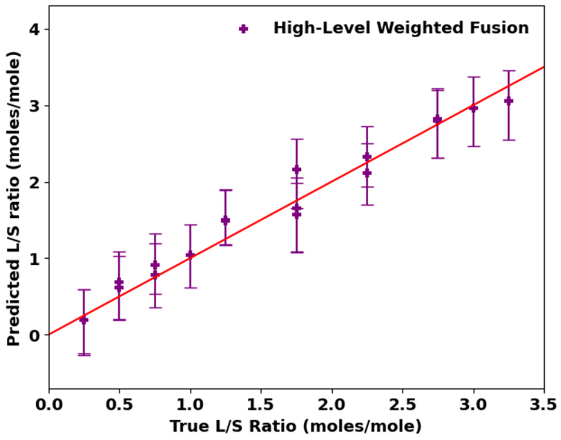
High-Level Weighted Fusion
Predicted L/S ratio (moles/mole)
True L/S Ratio (moles/mole)

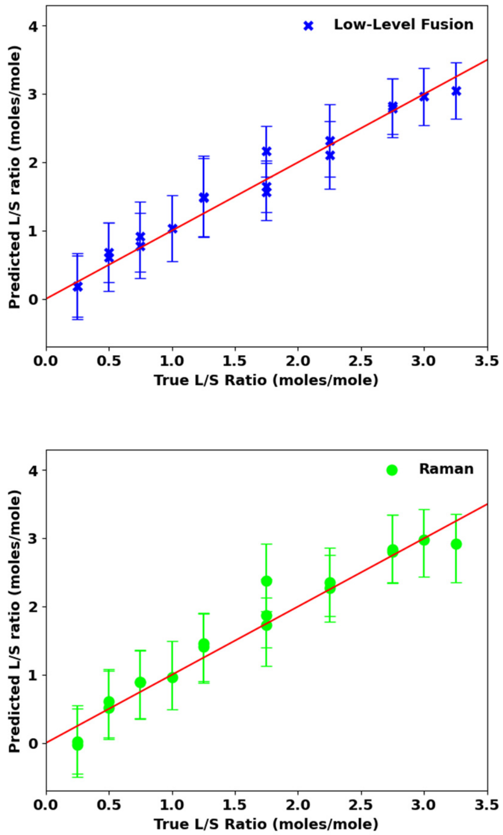

Figure S3. Test set performance of different models. The red line indicates the ideal prediction for each L/S ratio across the tested range. The absence of points around L/S of 1.5 is due to the absence of this data point in the test set, due to the random nature of the train/test split.

Disclaimer/Publisher's Note: The statements, opinions and data contained in all publications are solely those of the individual author(s) and contributor(s) and not of MDPI and/or the editor(s). MDPI and/or the editor(s) disclaim responsibility for any injury to people or property resulting from any ideas, methods, instructions or products referred to in the content.
